# Comparative Analysis of the Accuracy and Robustness of the Leap Motion Controller 2

**DOI:** 10.3390/s25247473

**Published:** 2025-12-08

**Authors:** Daniel Matuszczyk, Mikel Jedrusiak, Denis Fisseler, Frank Weichert

**Affiliations:** Department of Computer Science VII, Technical University Dortmund, 44227 Dortmund, Germany; daniel.matuszczyk@tu-dortmund.de (D.M.); mikel.jedrusiak@tu-dortmund.de (M.J.); denis.fisseler@tu-dortmund.de (D.F.)

**Keywords:** human–machine interaction, leap motion controller, measurement

## Abstract

Along with the ongoing success of virtual/augmented reality (VR/AR) and human–machine interaction (HMI) in the professional and consumer markets, new compatible and inexpensive hand tracking devices are required. One of the contenders in this market is the Leap Motion Controller 2 (LMC2), successor to the popular Leap Motion Controller (LMC1), which has been widely used for scientific hand-tracking applications since its introduction in 2013. To quantify ten years of advances, this study compares both controllers using quantitative tracking metrics and characterizes the interaction space above the sensor. A robot-actuated 3D-printed hand and a motion-capture system provide controlled movements and external reference data. In the central tracking volume, the LMC2 achieves improved performance, reducing palm-position error from 7.9–9.8 mm (LMC1) to 5.2–5.3 mm (LMC2) and lowering positional variability from 1.3–2.2 mm to 0.4–0.8 mm. Dynamic tests confirm stable tracking for both devices. For boundary experiments, the LMC2 maintains continuous detection at distances up to 666 mm, compared to 250–275 mm (LMC1), and detects hands entering the field of view from distances up to 646 mm. Both devices show reduced accuracy toward the edges of the tracking volume. Overall, the results provide a grounded characterization of LMC2 performance in its newly emphasized VR/AR-relevant interaction spaces, while the metrics support cross-comparison with earlier LMC1-based studies and transfer to related application scenarios.

## 1. Introduction

**Figure 1:**
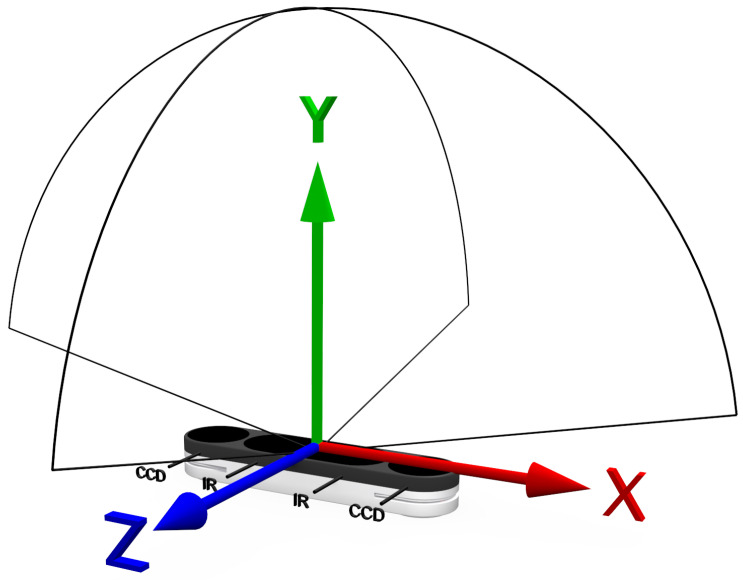
Schematic view of the LMC2 with the coordinate system and a $160^{\circ }\times 160^{\circ }$ field of view.

**Figure 2:**
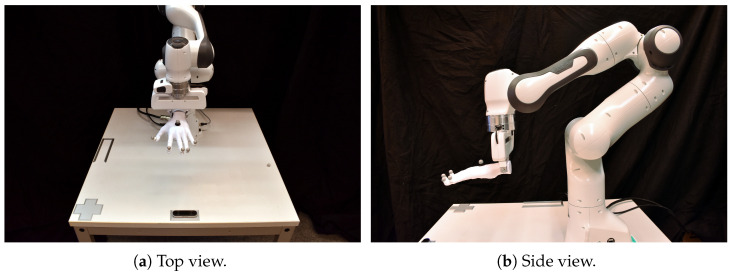
3D-printed hand attached to the Franka Emika Panda robot, (**a**) top view and (**b**) side view. Markers are attached to specific hand locations. The LMC’s position is either central or at the corner placed in a 3D-printed mount.

Upon the introduction of the Leap Motion Controller (Ultraleap Inc. https://www.ultraleap.com (accessed: 21 November 2025)) (LMC, the first-generation device is referred to as LMC1 and the second-generation device as LMC2 throughout this paper), the market for human–machine interaction (HMI) sensor development experienced significant activity, which also spurred a multitude of advancements in applications including industrial tasks, motion analysis, and gesture-based user interfaces [1]. In recent years, touchless hand-based HMI has evolved into the field of virtual reality (VR) and augmented reality (AR) applications. One example of this is the development of hand-tracking systems that are integrated directly into head-mounted displays and replace physical hand controller input devices. VR gesture recognition is attracting great attention among HMI fields [2]. Hence, the investigation of HMI technologies should align with VR/AR requirements, though without limiting itself to them alone. With the release of the Leap Motion Controller 2 (LMC2), depicted in Figure 1, a new device is competing in the growing field of VR/AR and HMI gesture interaction. The design of the updated version is smaller while providing a greater field of view for hand detection. The sensor is designed for different tasks like hand tracking, gesture-based user interfaces, and VR/AR scenarios. As the prior version of the LMC2 is used in many tasks, studies, and investigations, the utilization of the newer version in those fields can be expected [3,4]. For a meaningful comparison with the prior model, aspects like resolution, precision, and repetition rates must be considered. Therefore, an analysis is required that examines the spatial range typically involved in hand gestures and various movement speeds. To our knowledge, an analysis of the LMC2 and its relationship to the LMC in those fields has not yet been conducted. Therefore, in this contribution, a study of the accuracy and robustness of the LMC2 compared to its predecessor is presented. The research question focuses on investigating the interaction space above the controller, which is crucial for VR/AR applications and robotic control. Due to the LMC2’s software limitation to actual hand tracking (instead of the tool tracking available for the LMC) and for increased repeatability, our setup employs a robot-moved 3D-printed hand (see Figure 2) to examine the LMC2’s hand-tracking capabilities. The paper’s main contributions are results from different experiments considering varying measurement characteristics like primary accuracy, robustness, and detection space, designed to compare the two controllers against each other. This allows us to relate the results of studies on the LMC to studies on the LMC2.

The main contribution of this paper is to quantify how the LMC2 compares to the LMC1 in terms of accuracy and repeatability across central and boundary regions of the tracking volume, providing a first comparison of both controllers, outlining how these differences align with emerging VR/AR and HMI use cases and how they can be related back to existing LMC1 studies. The paper is structured as follows: After the introduction, related work is summarized in Section 2. This is followed by an overview of the experimental environment (Section 3) and a description of the conducted experiments (Section 4). Finally, the results are presented in Section 5 and discussed in Section 6.

## 2. Related Work

In the following section, an overview of recent developments in hand-tracking technologies for HMI, VR/AR, and gesture-based interfaces is provided, with a particular focus on studies involving the LMC. While numerous works have evaluated the original LMC1 in isolation, the literature still lacks a comparison between LMC1 and its successor. This gap is central to the motivation of the present study, which aims to enable a direct performance mapping between both generations under identical, controlled conditions, so that the subsequent experiments can be interpreted within the broader evolution of hand-tracking technology. The related work further encompasses general applications of 3D sensing technologies, such as the Kinect (Microsoft Corporation, https://www.microsoft.com (accessed: 21 November 2025)) and the Leap Motion Controller, as well as specific research topics in hand-gesture detection, sign-language recognition, and healthcare [5]. Considering these investigations provides the fundamentals for outlining a test scenario that covers the relevant parameters and use cases for the present study and enabling a systematic comparison between the LMC1 and the LMC2.

Due to their suitability for VR and AR applications, LMCs have been investigated in various surveys and articles. Aspects for analysis are device comparison, accuracy, precision, existing gesture recognition algorithms, and the price of the devices [5]. Furthermore, the LMC’s accuracy has been investigated in several articles. In one of the first analyses, the accuracy, robustness and repeatability were evaluated by using an industrial robot holding a pen to set up a reference system [1]. With this setup, a single LMC was analyzed in static and dynamic scenarios in order to obtain benchmark parameters for human-computer interaction. Subsequent studies built upon this baseline and further examined accuracy and robustness to identify the Leap sensor’s suitability for static and dynamic motion capture, fingertip tracking, and gesture classification tasks [3,6,7,8]. Additionally, the LMC’s pointing task accuracy has been researched in VR environments and even fingertip gesture recognition for VR interactions was investigated [4,9]. In addition, the latest LMC2 has been leveraged in portable, head-mounted configurations to enable AR/VR hand tracking, demonstrating the controller’s feasibility for mobile HMI in daily use [10]. More recent accuracy analyses report characteristic error patterns for LMC1, e.g., increased distortion and instability towards the edges of the tracking volume [11]. Furthermore, typical use cases for the LMC are hand gesture detection, sign language recognition, HMI, and healthcare applications [5]. Multiple studies have examined these areas in depth, highlighting different perspectives and findings. In the case of sign language recognition using the LMC, this includes the identification of different languages, for example Australian sign language, Chinese sign language, American sign language, and many others, also in real time and air-writing techniques [12,13,14,15,16]. During the sign language recognition studies, a box-shaped volume of about 40 cm side length for the hand detection has been identified, with detection accuracy decreasing close to the box corners [12]. To overcome occlusion and field-of-view limitations in such hand gesture scenarios, multi-view approaches have recently been introduced for the LMC2. For instance, a dual-LMC2 setup was used to capture a comprehensive dataset of hand poses from multiple angles [17]. However, the effective operating space of a single LMC2 device without dual-view augmentation still remains to be systematically investigated, which is of particular relevance for sign language recognition use cases where reliable tracking across the entire interaction volume is required.

For HMI tasks, the LMC’s capability has been analyzed in terms of 3D interaction scenarios controlling robots, especially adaptive robot arm manipulation [18,19,20]. Moreover, recent work integrated the LMC2 into collaborative robotic environments to achieve intuitive gesture-based robot control, further highlighting its usefulness for human-robot interaction [21]. Furthermore, the Leap Motion Controller’s capabilities have been investigated under the use case of hand gesture recognition, with even analyses for the LMC2 [22,23,24]. However, the LMC2-related work focuses on gesture-dataset generation and recognition performance rather than on quantitative tracking accuracy. Consequently, this work reports no 3D positional errors, region-dependent distortions, or repeatability measures for the LMC2.

Another operational area for the LMC is the healthcare domain, where stroke rehabilitation in particular is a major field of research [25]. For this, the device is used for free-hand interactions and hand gesture recognition for post-stroke rehabilitation [26,27]. However, the usage is not limited to only stroke rehabilitation but also allows evaluation and assessment of upper limb motor skills in Parkinson’s disease [28]. Especially healthcare analyses impose stringent requirements on accuracy, which must be carefully accounted for in upcoming test cases. In recent VR/AR and medical-interaction studies, modern hand-tracking systems are increasingly used in practical scenarios such as VR stylus interaction [29], projected rehabilitation games [30], or medical imaging visualization workflows [31]. These works underline the growing importance of reliable markerless hand tracking across different application domains. However, they focus on task performance and interaction design rather than on detailed, quantitative tracking assessment. Alongside Leap Motion devices, alternative hand-tracking solutions have gained traction. MediaPipe (https://ai.google.dev/edge/mediapipe/ (accessed: 21 November 2025)) Hands, Azure Kinect, and Intel RealSense (https://realsenseai.com/ (accessed: 21 November 2025)) represent camera-based or depth-based pipelines with different accuracy-latency trade-offs. A recent study compares the LMC2 with MediaPipe in a human motion-capture task and reports task-level differences in joint-angle and trajectory estimates [32]. While such studies highlight the practical relevance of comparing different tracking systems, the use of human motion as reference introduces natural movement variability and does not yield metrology-grade 3D error maps or repeatability metrics. Markerless clinical-assessment studies [33] and comparative analyses of marker-based and markerless systems [34] likewise demonstrate that algorithmic alternatives may perform well in specific scenarios, yet none provide a structured 3D error characterization across the entire interaction volume comparable to metrology-style Leap Motion evaluations.

Despite extensive prior work on the original Leap Motion Controller and a growing number of application studies involving LMC2, a quantitative, head-to-head comparison of both generations using common metrology-style metrics is still missing. In particular, previous LMC1 evaluations report heterogeneous accuracy values and spatial error maps that have not been revisited with LMC2, and existing LMC2-based applications do not provide detailed error characterizations. The present study addresses this gap by aligning its evaluation with established accuracy and repeatability measures [1,11] and by explicitly contrasting LMC2 with LMC1 across shared, robot-actuated test conditions. By characterizing central and boundary regions of the tracking volume using an external motion-capture reference, the results enable a quantitative assessment of ten years of Leap Motion hardware evolution, a re-interpretation of earlier LMC1-based findings in the light of LMC2 performance, and a clearer positioning of LMC2 within the current ecosystem of VR/AR hand-tracking technologies.

In the following, we therefore move from this literature-based gap analysis to the experimental design and measurement setup (Section 3) that provide the required quantitative accuracy and repeatability metrics for both controllers.

## 3. Materials and Methods

To address the research question and to enable a head-to-head comparison between LMC1 and LMC2, this section introduces the robot-based measurement setup, the reference system, and the metrology used to quantify accuracy, repeatability, and detection range relevant for VR/AR applications and other use cases. Therefore, experiments for static and dynamic test cases covering these aspects need to be conducted in order to compare the LMC and the newer LMC2. For this, a static setup is arranged to ensure a consistent environment during different tests. The setup consists of the two LMC with their specifications described in Section 3.1. To guarantee comparable hand tracking, 3D-printed hands, described in Section 3.2, are used. In combination with a Franka Emika Panda (Franka Robotics GmbH https://franka.de/ (accessed: 21 November 2025)) robot arm (Section 3.3), a reproducible hand movement during repetitions and experiments is achieved. In order to obtain a comparable solution, a reference tracking system is used for the measurement setup.

### 3.1. Leap Motion Controller

Similar to its predecessor, the LMC2 is designed for hand gesture and finger position detection. A schematic view of the controller is presented in Figure 1. Due to the usage of two Charge-Coupled Device (CCD) cameras and two infrared emitters, both controllers observe and determine hand positions above the controller. The newer version has a higher resolution and a greater field of view, being even smaller and lighter than the first version (distance up to 110 cm, across $160^{\circ }\times 160^{\circ }$ maximum field of view). For the experiments, the same software stack for both controllers was used to ensure comparability (Ultraleap Tracking Service 5.12.0 (Gemini runtime) with identical configuration and gesture settings for LMC1 (firmware version 1.7.0) and LMC2 (firmware version 3.8.7)). This guarantees that observed differences between devices stem from hardware and internal tracking improvements rather than from differing software configurations. Due to a limitation in the LMC2’s software compatibility, the tool-tip detection mode is unavailable. This mode, which was supported in earlier software versions, allows the LMC to detect only the tip of a stylus or similar tool, enabling highly precise positional tracking. This limitation affects the experiments, since a setup for measuring accuracy parameters using a tool, as done in other studies, is not feasible [1].

### 3.2. 3D-Printed Hand

For the experiments, a 3D-printed hand is used in order to have reliable measurement relations. Therefore, the hand is designed to capture real hand dimensions, its length 17.5 cm (finger to wrist) and width 12 cm similar to other studies where self-made hands were used [35]. The hand is printed in two parts, the hand as itself and a wrist. The wrist is printed with high infill density and the hand with low infill density (i.e., the amount of internal material used during 3D printing) in order to ensure a center of mass next to the robot gripper. A dovetail joint ensures a rigid connection between the two parts. 3D-printed conic grippers with notches are used for joining the wrist with the Franka hand. This results in a firm centered grip, minimizing wobbling during robot motion. Figure 2 shows a left hand in use. A left and a right hand were constructed for the experiments, with both hands being mirrored copies of each other in order to maintain comparability.

### 3.3. Measurement Setup

As an additional source of continuous external reference measurements, an OptiTrack (NaturalPoint Inc. https://www.optitrack.com/ (accessed: 21 November 2025)) tracking system with sub-millimeter accuracy is utilized (calibrated with the standard OptiTrack wanding procedure, reporting a mean ray error of 0.707 mm and a mean wand error of 0.115 mm for a volume of approximately 3.5×4×2 m), providing independent data for validation purposes. The system consists of twelve infrared cameras with a centered view on the experiment setup with the controllers and the 3D-printed hand attached to the robot arm (Figure 2). With the infrared cameras, an observation of marker-based rigid bodies is achieved. For this, markers are attached to the 3D-printed hand at each fingertip and at the palm position (Figure 2b). The coordinate system for the OptiTrack tracking data is set directly next to the Leap Motion device. The hand’s positional data is streamed and combined with the LMCs data via nearest timestamps, since the OptiTrack measurement provides a higher frame rate. Although nearest-timestamp matching can have small temporal inconsistencies, this effect is mitigated in our precision experiment (Section 4.2). Here, the hand was static for a defined holding and settling period before each measurement, ensuring that a stable pose was maintained during data capture. This controlled setup substantially reduces the impact of latency fluctuations between the OptiTrack and LMC streams. Fixed positions for the Leap Motion Controllers are maintained using 3D-printed holders designed for each specific location. The 3D-printed holders ensure reproducible and a fixed sensor placement relative to the Franka Emika Panda base coordinate frame, where the x-axis points forward, the y-axis to the left, and the z-axis upward. The centrally mounted LMC holder is positioned such that its sensor center is located 43.6 cm along the x-axis from the robot base frame origin. The outer controller is mounted with a lateral offset of 35.2 cm along the y-axis and a forward offset of 42.1 cm along the x-axis. This ensures that each controller is placed in exactly the same position across all experiments (see Figure 2). During all robot-assisted measurements, the Franka Emika Panda (Franka Robotics GmbH https://franka.de/ (accessed: 21 November 2025)) was operated using the velocity scaling interface provided by the Franka Control framework. In this interface, motion commands are executed relative to the robot’s internally defined dynamic limits, where a scaling of 100 % corresponds to the maximum joint and Cartesian velocities as specified by the manufacturer (approximately 2 rad/s joint velocity and 1.7 m/s Cartesian end-effector velocity). A scaling of 50 % therefore results in movements at half of these velocity limits while maintaining the same motion profiles and control laws. This relative scaling ensures reproducible robot motion across experiments. Potential sources of experimental error include residual robot vibrations after stopping at a grid position, partial occlusions of markers in extreme poses, and small timing offsets between the OptiTrack and Leap Motion data streams. We mitigated these effects by operating in a completely darkened lab to avoid disturbing infrared light, by using rigid 3D-printed mounts for the controllers and the hand, by briefly pausing the robot at each grid point, and by synchronizing the data via nearest timestamps. Remaining uncertainties are reflected in the reported repeatability values and are further discussed in Section 6.

### 3.4. Metrology

In order to describe and evaluate the captured data, the following mathematical formalizations are used [1]. In metrology terms, accuracy describes how close the mean measured position is to the OptiTrack reference, whereas repeatability describes how tightly repeated measurements cluster around their mean at a fixed position. We distinguish between the reference points ${\tilde{p}}^{\left[i,j\right]}=\left({\tilde{p}}_{x}^{\left[i,j\right]},{\tilde{p}}_{y}^{\left[i,j\right]},{\tilde{p}}_{z}^{\left[i,j\right]}\right)$ in relation to the coordinate system of the OptiTrack and the registered points $p^{\left[i,j\right]}=\left(p_{x}^{\left[i,j\right]},p_{y}^{\left[i,j\right]},p_{z}^{\left[i,j\right]}\right)$ of the Leap devices. In this context, i∈N represents the approached position mapped to the natural numbers and j∈N the repetition performed at the same position. We denote the arithmetic mean $\tilde{\mu }$ of the measured points for the reference system at a fixed position *i* by

(1) $$\begin{array}{c} {\tilde{\mu }}^{\left[i\right]}=\frac{1}{N}\sum_{j=1}^{N}{\tilde{p}}^{\left[i,j\right]}. \end{array}$$

In general, we avoid the index *j* when averaging over the number of repetitions. The formalization applies analogously to the local systems of the Leap devices. Based on this, we define the accuracy with regard to the OptiTrack system by

(2) $$\begin{array}{cc} Acc^{\left[i\right]} & =∥{\tilde{\mu }}_{h}^{\left[i\right]}-T_{h}\mu_{h}^{\left[i\right]}{∥}_{2} \end{array}$$

(3) $$\begin{array}{cc} Acc & =\frac{1}{P}\sum_{i=1}^{P}Acc^{\left[i\right]} \end{array}$$

where P∈N is the number of positions and $T_{h}\in R^{4\times 4}$ is the transformation matrix between the coordinate systems of the OptiTrack system and the Leap devices. Homogeneous vectors are indexed by an *h*. To enable a valid comparison between the Leap Motion data and the OptiTrack reference system, a calibration of the coordinate systems was carried out prior to the evaluation. Corresponding fingertip and palm positions are sampled across the full 3D grid. From this dataset, only geometrically consistent and stable position samples are retained, yielding m≥64 calibration points in the full grid and m=27 in the focus region. For each calibration point, representative Leap Motion and OptiTrack positions are obtained by averaging over a short stationary interval around the target pose, while removing noisy samples based on a robustness criterion. The residual alignment error of the resulting rigid SVD fit was approximately 13 mm over the full grid and 8 mm within the central focus region (median values), reflecting the known increase in Leap Motion positional distortions toward the periphery of the tracking volume. Following this, we determine our transformation using a rotation matrix $R\in R^{3\times 3}$ derived from the singular value decomposition (SVD) [36] and a matching translation vector $t\in R^{3}$, which satisfies the conditions

(4) $$\begin{array}{cc} \; & \min_{R}\sum_{i=1}^{m}{∥\left({\tilde{p}}^{\left[i\right]}-{\tilde{\mu }}^{\left[i\right]}\right)-R\left(p^{\left[i\right]}-\mu^{\left[i\right]}\right)∥}^{2} \end{array}$$

(5) $$\begin{array}{cc} \; & \min_{t}\sum_{i=1}^{m}{∥{\tilde{p}}^{\left[i\right]}-Rp^{\left[i\right]}-t∥}^{2}. \end{array}$$

Alongside the accuracy, we also determine the standard deviation

(6) $$\begin{array}{c} \sigma^{\left[i\right]}=\sqrt{\frac{1}{N}\sum_{j=1}^{N}{\left(p^{\left[i,j\right]}-\mu^{\left[i\right]}\right)}^{2}}. \end{array}$$

in order to evaluate the repeatability of the positioning.

## 4. Experiments

We evaluate both Leap Motion devices in terms of their stability during continuous motion within the detection domain, their accuracy to track repeatedly approached positions and their behavior at the domain boundary. In order to analyze and compare the two controllers we conduct three different experiments (A continuous experiment, B field of view experiment, C boundary experiment), which are described in the following sections. The experiments are performed one after another, with a 3D-printed left or right hand, which is mounted on a robotic arm in order to take hand-side effects into account. Each finger and the palm are equipped with individual markers that are tracked by an OptiTrack system, which has been used as a reference system for our study. Each experiment was carried out 10 times in a completely darkened room to prevent side effects related to the infrared technology used and solar radiation. The choice of ten repetitions per condition follows established practice in metrology, where repeatability is commonly estimated from multiple identical measurements and where ten repetitions are generally sufficient to obtain stable standard deviation estimates under controlled conditions. Comparable numbers of repetitions have also been used in related Leap Motion validation studies. For example, Vysocký et al. [11] employ repeated robot-actuated movements to assess Leap Motion stability, and Sprague et al. [32] evaluate the Leap Motion Controller 2 and MediaPipe Hands using five repetitions per hand-movement task. Moreover, preliminary observations in our own setup consistently showed highly similar measurements across repeated runs. Therefore, ten repetitions represent a statistically sound and practically efficient choice for achieving stable estimates of accuracy and repeatability in our controlled measurement setup.

### 4.1. Continuous Experiment

**Figure 3:**
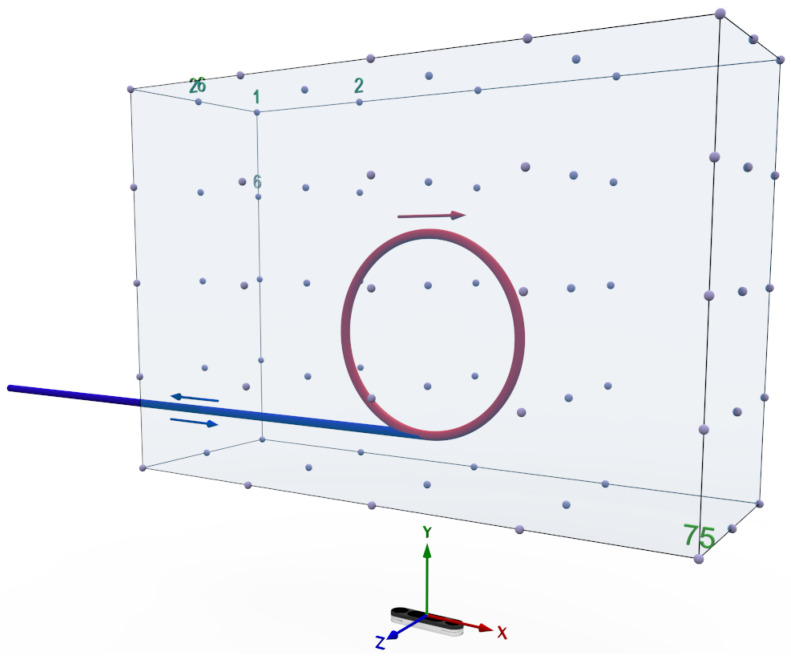
The three distinct experiments result in three different motion patterns of the robot: continuous circular motion (red circle) and a 75 point grid to be traversed, both located directly above the controller, and a trajectory extending from the center above the controller outwards and vice versa (blue line).

In our continuous experiment (A), we investigate the accuracy for the Leap Motion controllers’ ability to capture a continuous non-linear circular hand movement above the controller. This test is essential to assess the tracking fidelity of the Leap Motion, particularly in dynamic and repetitive scenarios, which are common in VR/AR applications. For this purpose, the Leap Motion is positioned along the vertical axis passing through the imaginary center of the circular motion, as illustrated in Figure 3. A robotic arm is used to ensure precise and reproducible hand movements, executing five circular movements above the LMC at a constant speed and a fixed radius of 5 cm, with the center of the circular trajectory located 45 cm along the x-axis and 30 cm along the z-axis from the robot base frame. This setup isolates variables such as hand speed and radius, allowing for controlled measurements of tracking performance. Specifically, the experiment measures two key performance indicators: (1) the failure rate, which captures the system’s ability to maintain continuous tracking of the hand without interruptions, and (2) positional accuracy, evaluated by the deviation of detected points when the hand repeatedly moves to the same position on the circular path. These metrics are critical for understanding the controller’s suitability for VR/AR scenarios, where high accuracy and reliability are non-negotiable. The results of this experiment are expected to reveal the strengths and limitations of the Leap Motion’s optical tracking system in detecting and following non-linear motions. A high failure rate or significant positional deviations could compromise the user experience, leading to inaccuracies in gesture recognition, unintended interactions, or user frustration. Conversely, robust performance under these test conditions would demonstrate the controller’s potential to enhance natural interaction techniques, contributing to a seamless and immersive VR/AR experience.

### 4.2. Field of View Experiment

**Figure 4:**
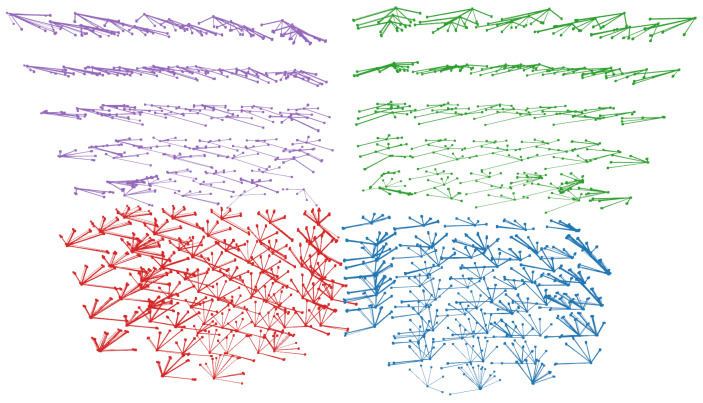
Visualization of the thumb, index, middle, ring, pinky, and palm positions detected by LMC1 for the left (red) and right (blue) hands, and by LMC2 for the left (purple) and right (green) hands.

In the field of view experiment (B), we examine the Leap Motion Controller’s accuracy when detecting hand positions within a predefined three-dimensional grid structure. This test evaluates the device’s accuracy and repeatability in stationary hand tracking, which is a critical aspect for tasks requiring fine motor control and steady input in VR/AR environments. The experimental setup involves traversing a series of fixed positions within a 3D grid, as illustrated in Figure 3. At each position, the robotic arm halts momentarily to minimize any residual vibration that could interfere with the measurements. The Leap Motion 2 then records the detected positional data for analysis. The primary goal of this experiment is to assess the maximum deviation in the device’s position detection during self-positioning. The predefined grid consists of 75 static target positions arranged in a regular 3×5×5 Cartesian structure. To ensure reproducibility of the experiment using a Franka Emika Panda robot, the grid is explicitly defined in the robot’s base coordinate frame. The three layers along the x-axis are located at 30 cm, 40 cm, and 50 cm, resulting in a spacing of 10 cm. Along the y-axis, five columns are sampled at −30 cm, −15 cm, 0 cm, 15 cm, and 30 cm, corresponding to a spacing of 15 cm. Along the z-axis, five rows are positioned at 15 cm, 25 cm, 35 cm, 45 cm, and 55 cm, with a spacing of 10 cm. This produces a uniformly structured sampling volume of 20×60×40 cm above the LMC. For each of the 75 grid points, the robotic arm moves to the specified coordinate and pauses to allow stable hand pose acquisition. The analysis considers two configurations: (i) the complete grid comprising 75 points, and (ii) a reduced cubic grid of 27 points, in which the top and bottom layers and the lateral side faces are excluded. The need for two levels of detail ((i) 75 points and (ii) 27 points) is visualized in Figure 4. To enable a valid comparison between the Leap Motion data and the OptiTrack reference system, a calibration of the coordinate systems was carried out prior to the evaluation. As outlined in Section 3, the transformation $T_{h}$ between both systems was determined by recording corresponding points for each finger and the palm while traversing the predefined grid. Within this interval, mean positions were computed both for the OptiTrack markers and for the Leap Motion detections. Outliers were removed using a median absolute deviation filter (c=3.5), resulting in robust mean positions for calibration. With these correspondences, the rigid transformation was estimated using SVD to obtain a rotation matrix R and translation vector t. This calibration ensured that the subsequent accuracy and repeatability analyses were performed in a consistent coordinate frame.

This metric reflects the Leap Motion’s capacity for maintaining stable and consistent tracking in static scenarios. Such scenarios are frequently encountered in VR/AR applications, for instance virtual object manipulation, where users expect precise and steady tracking of their hand or fingers while interacting with digital elements. For VR/AR systems, stability in stationary tracking is as important as dynamic gesture recognition. Inaccurate or fluctuating positional data could lead to misalignment of virtual objects, reduced immersion, or difficulties in performing delicate tasks like virtual drawing or assembling. By systematically measuring deviations across a structured grid, we aim to identify regions within the Leap Motion’s detection space that may exhibit reduced accuracy or increased variability. Additionally, this experiment highlights potential limitations of the Leap Motion 2’s optical tracking technology when dealing with static inputs. Such insights are particularly valuable for applications requiring high precision, such as virtual medical training, or VR/AR interfaces for professional workflows. Through this field of view experiment, we provide a focused evaluation of the Leap Motion’s static tracking accuracy and its ability to perform under controlled and repeatable conditions. Together with other dynamic tracking tests, this experiment contributes to a comprehensive understanding of the Leap Motion’s robustness, accuracy, and potential for VR/AR applications.

### 4.3. Boundary Experiment

In order to evaluate the Leap Motion’s potential for real-world applications, we analyzed its tracking performance at the boundaries (C) of its detection domain. This experiment focuses on the device’s ability to seamlessly detect and track a hand as it enters and exits its field of view. Such edge-case scenarios are particularly relevant for VR/AR applications, where users frequently move their hands in and out of the interaction space, whether intentionally or unintentionally. The experiment comprises two cases: a robotic arm guiding a hand from outside the detection domain into the LMC’s field of view, and the reverse movement from within the field of view to the outside, as depicted in Figure 3. The primary focus is on two performance metrics: (1) the time required for the LMC to detect the hand as it enters the field of view, and (2) the accuracy and stability of tracking at the edges of the detection space. To ensure full reproducibility, the robot follows a strictly linear trajectory along the y-axis of the Franka Emika Panda base frame. The movement covers a total distance of 68 cm, moving from −34 cm outside the LMC’s field of view to a position of 34 cm along the y-axis inside (and vice versa) with an x-axis offset 40 cm from the robot base and a height of 20 cm above the sensor. This controlled linear movement ensures that only the entry and exit behavior of the Leap Motion is tested. These metrics are critical for understanding how the device handles transitions between detected and undetected states, which is an important consideration for seamless and immersive VR/AR experiences. In practical applications, edge behavior can significantly impact user experience. For instance, delayed detection when a hand re-enters the field of view could result in lag or missed gestures, disrupting the fluidity of interactions. Similarly, inaccuracies or tracking instability near the boundaries could lead to misinterpreted gestures or unintended interactions. By quantifying these behaviors, the experiment identifies potential limitations and edge-related challenges for the Leap Motion in VR/AR systems.

## 5. Results

To enable a clear comparison between LMC1 and LMC2 and to assess their suitability for VR/AR and other HMI applications, the results are structured according to the three experiment types. Section 5.1 contains the results of the experiments for the continuous circular movement (A). The results for the field of view experiment (B) are presented and elaborated in Section 5.2, analogous findings for the boundary experiments (C) are detailed in Section 5.3.

### 5.1. Continuous Test Cases

**Figure 5:**
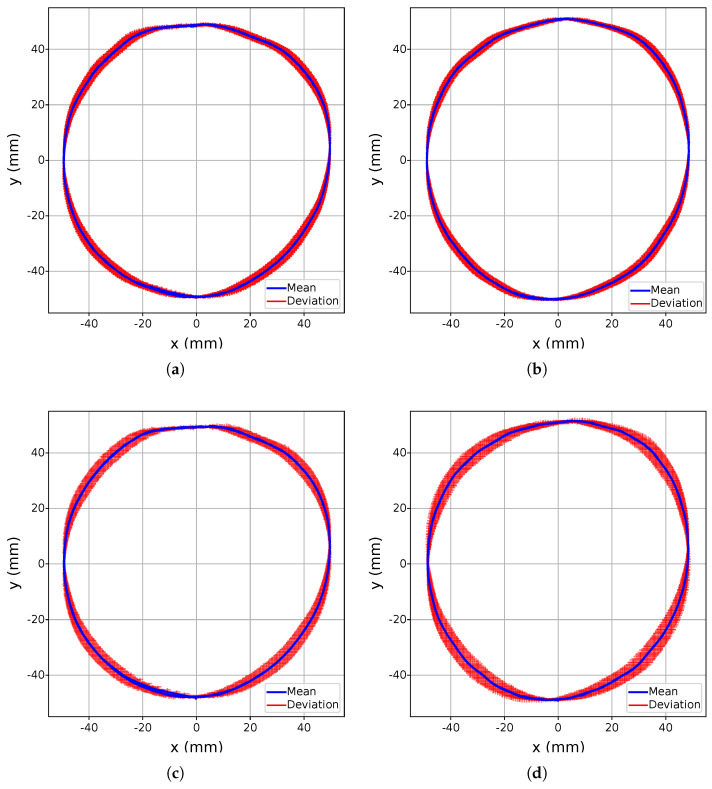
Circular movement evaluation of the LMC1 in the xy-plane. The average trajectory is shown in blue, while the standard deviation across all repetitions is indicated in red. The execution at 50% speed is depicted for the left hand in (**a**) and for the right hand in (**b**), while the execution at 100% speed is shown for the left hand in (**c**) and for the right hand in (**d**).

**Figure 6:**
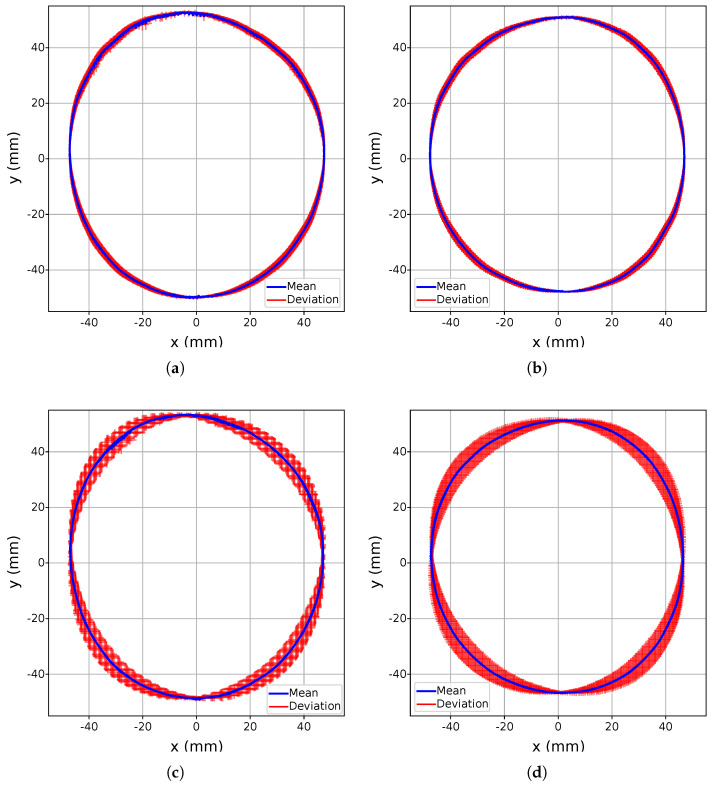
Circular movement evaluation of the LMC2 in the xy-plane. The average trajectory is shown in blue, while the standard deviation across all repetitions is indicated in red. The execution at 50% speed is depicted for the left hand in (**a**) and for the right hand in (**b**), while the execution at 100% speed is shown for the left hand in (**c**) and for the right hand in (**d**).

The primary objective of the continuous movement experiment (A) was to evaluate the stability of the Leap Motion 2’s tracking within its detection domain, specifically identifying any potential deviations or inconsistencies in the recorded data. The results, visualized in Figure 5 and Figure 6, highlight the standard deviation of the detected points across the coordinate planes xy, with deviations marked by red crosses. Analysis reveals that outliers remain within the millimeter range, demonstrating a high degree of stability and precision throughout the experiment. Notably, there were no interruptions in detection during continuous hand movements, underscoring the robustness of the LMC2’s tracking capabilities under controlled conditions. When comparing LMC2 to its predecessor, a consistent, albeit minor, deviation in the vertical axis (*z*) was observed during repeated movements. This vertical drift, while small, suggests that certain systematic factors, potentially related to the updated sensor positioning or processing algorithms, may contribute to subtle variations in tracking accuracy. Nevertheless, the magnitude of the standard deviation is comparable between the two generations, as illustrated in Figure 5 for LMC1 and Figure 6 for LMC2, suggesting that overall tracking stability remains at a similar level despite generational differences. An increased execution speed, however, results in larger deviations, as evidenced by the more pronounced dispersion of the red markers. From an application perspective, the millimeter-level variability observed in the circular motion paths suggests that both devices can support smooth mid-air pointing and gestural input in VR/AR scenarios, as long as interaction techniques are designed to be robust against small trajectory perturbations rather than relying on exact path reconstruction.

### 5.2. Field of View Experiment

The field of view experiment (B) is designed to assess both recognition and positional accuracy within a defined three-dimensional detection space above the Leap Motion sensors. This evaluation is essential in order to understand the spatial limitations and strengths of the device, particularly for VR/AR applications requiring precise hand tracking throughout a diverse interaction volume. As represented in Table 1, the detection and accuracy performance of both the LMC1 and its successor (LMC2) exhibit a consistent pattern for all 75 points. For comparison, Table 2 presents the performance values of both controllers within the reduced cubic grid of 27 points, focusing on the area directly above the controller. Lower values clearly indicate a more precise hand recognition directly in the center above each Leap Motion Controller.

Additionally, Table 3 illustrates the number of failed detections and successful measurements for the respective configurations. A failed detection refers to a complete loss of hand tracking, i.e., no valid data for the palm or any finger was returned by the Leap Motion device for the corresponding grid position. Partial detections (e.g., missing individual fingers) did not occur in our setup. The device either provided a full hand pose or no pose at all. This binary behavior is consistent with the internal confidence model of the tracking pipeline, which rejects low-confidence or geometrically inconsistent estimates instead of providing degraded partial outputs. The success rate of 100% in the 27-point core grid is therefore explained by the fact that all positions in this central region lie well within the optimal field of view, where the device maintained continuous and reliable full-hand detection for all repetitions and for both controller generations. With respect to accuracy, the results indicate an improvement of LMC2 compared to its predecessor. However, the larger field of view appears to affect repeatability: the hand is detected more often, but the positional values deviate more strongly. When the evaluation is restricted to the central focus area of 27 points, a clear improvement from LMC1 to LMC2 becomes evident. In combination with the other tables and with Figure 4, this indicates that both accuracy and repeatability decrease towards the peripheral regions of the field of view, and that particularly at the very edges a noticeable distortion of the detected hand occurs. However, when taking the palm position as a robust indicator of the overall hand tracking performance, the results still reveal improved accuracy and stability for the LMC2 compared to the LMC1, despite the distortions observed at the periphery of the field of view.

**Figure 7:**
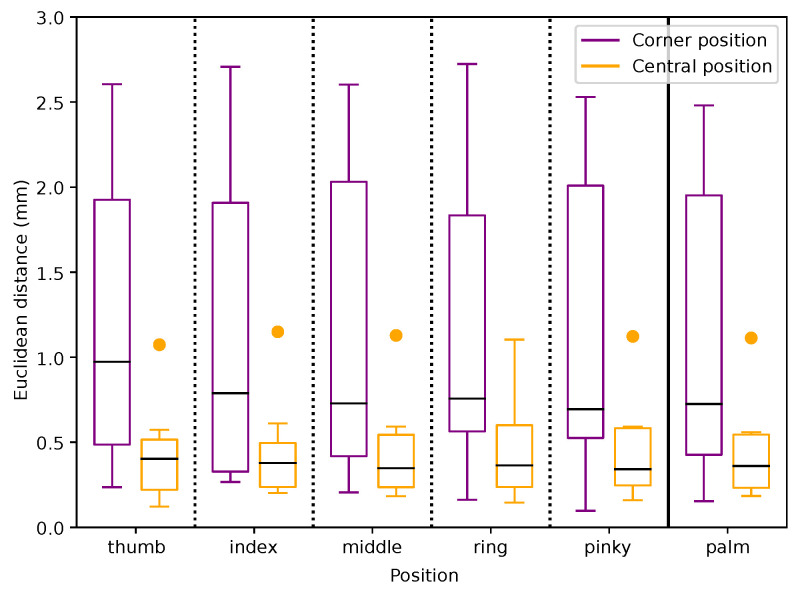
Euclidean distance from obtained reference points for the fingers and palm positions on the LMC2. The purple boxplots are for a corner point (point 1) and the orange boxplots are from a point (point 43) above the Leap Motion Controller (see Figure 3).

**Figure 8:**
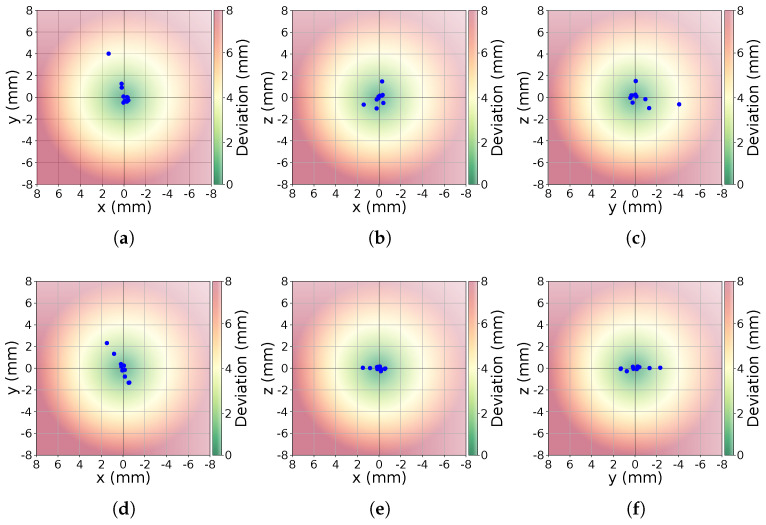
Deviation in mm with respect to the reference point in the field of view experiment at index position 29. For the coordinate planes xy, xz and yz the results are presented in (**a**–**c**) (LMC1) and (**d**–**f**) (LMC2).

In the two-dimensional projections of the detection space, a central region directly above the sensor shows significantly better accuracy and recognition rates. However, both metrics degrade noticeably as the hand approaches the boundaries of the detection domain. This degradation is observed for all axes (xy, xz, yz), emphasizing the challenges in achieving reliable tracking near the edges of the sensor’s field of view. This central-to-boundary performance gradient is further corroborated by the boxplot analysis in Figure 7. Furthermore, a region-wise analysis of the 75-point field of view grid quantifies changes from the central interaction space toward the edges. The grid is partitioned into three defined regions. The 27 points of the inner 3×3×3 cube directly above the sensor (center), the surrounding non-corner points (middle ring), and the eight outermost vertices of the grid (corner). Aggregating all fingers and both hands, the LMC1 has a median Euclidean error of 10.9 mm in the center region, increasing to 18.9 mm in the middle ring and 25.7 mm at the corners. For LMC2, the corresponding median errors are 9.4 mm (center), 14.2 mm (middle ring), and 32.4 mm (corners), indicating an improvement over LMC1 in the central and mid-range volume but similar deviation at the edges of the field of view. For the LMC1, the median values increase from (|Δx|,|Δy|,|Δz|)=(5.1,7.0,2.9) mm in the center to (14.2,19.5,5.3) mm in the corners. Corresponding for the LMC2 from (5.4,3.6,3.1) mm to (23.2,8.3,7.0) mm. The deviations |Δz| remain comparable smaller for both devices across all regions. Quantitatively, the reduced 27-point core region exhibits Euclidean errors in the one-digit millimeter range and markedly smaller dispersion than the outer ring of points, whereas positions near the corners of the 75-point grid show clearly increased errors and wider spread. This confirms that LMC2’s main performance gain over LMC1 is achieved in the central interaction space, while both devices share a similar qualitative degradation pattern towards the boundaries of the tracking volume. For a hand positioned at the center of the detection domain (orange boxplot), the deviation in recorded positions for both fingers and palm remains minimal, with most measurements clustering tightly around the expected values. In contrast, at a position located near the upper left corner of the detection box (purple boxplot), deviations are significantly larger, indicating reduced tracking precision. This pattern highlights a key limitation of the Leap Motion technology: while highly accurate in its central detection space, its reliability diminishes closer to the edges. In general, with regard to the accuracy and repeatability of the measurements, an improvement is evident in the LMC2 compared to its predecessor, as can be seen in Table 1. This is also confirmed by the Figure 8, which indicates a smaller deviation. Especially regarding the xz- and yz-coordinate plane, the variance is reduced compared to LMC1. In practical terms, palm errors in the central 27-point region on the order of 5–10 mm with sub-millimeter repeatability indicate that LMC2 is well suited for mid-air selection, menu interaction, and manipulation tasks in VR/AR. At the same time, the substantially larger errors and variability near the volume boundaries emphasize that interaction techniques should keep critical actions within the central region whenever possible. Because the qualitative degradation pattern from center to corners is similar for both generations, existing LMC1-based application studies can still be interpreted in a comparable way, while our results suggest that LMC2 will typically reduce absolute position errors by a few millimeters in the central interaction space.

### 5.3. Boundary Experiment

In contrast to the experiments in Section 5.1 and Section 5.2, the boundary experiment (C) specifically examines the LMC1 and its successor (LMC2) detection boundaries as they track the hand entering and exiting their detection domain. This test is critical for understanding how well the devices handle transitions at the edges of their detection zone, a scenario frequently encountered in VR/AR applications where user interactions often begin or end at the limits of the interaction space. Table 4 and Table 5 depict the differences between entering and leaving the detection space. The results demonstrate that the LMC2 covers a larger field, both in terms of prolonged detection when the hand moves outward and in earlier recognition when the hand enters the field of view. This leads to a maximum continuous detection distance of 666.28 mm and the earliest detection at 646.52 mm. Furthermore, the choice of hand appears to influence how early and how long the detection is maintained. Considering these values, movements away from the sensor result in a longer detection range compared to movements approaching the sensor from outside its field of view.

**Figure 9:**
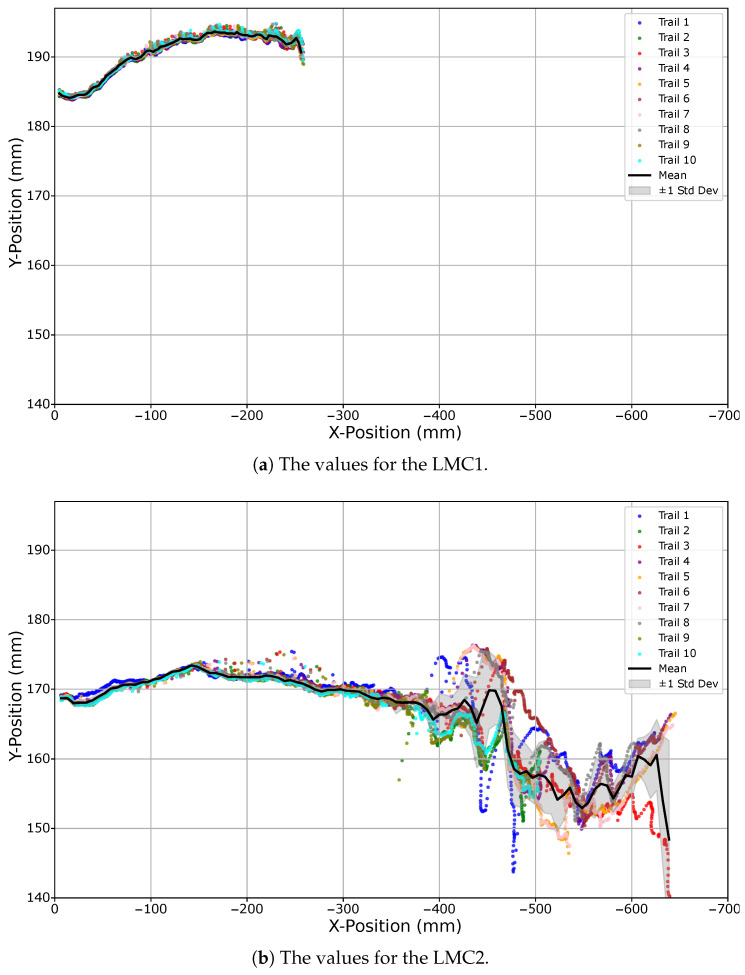
Boundary experiment results for movements from above the LMC to outside its detection field. Panels show executions with the right hand at 50% robot speed: LMC1 in (**a**) and LMC2 in (**b**). Each dotted line represents one of ten trials, the black line indicates the mean trajectory, and the gray area denotes the standard deviation. Identical axis scaling is used to enable direct comparison of detection performance.

**Figure 10:**
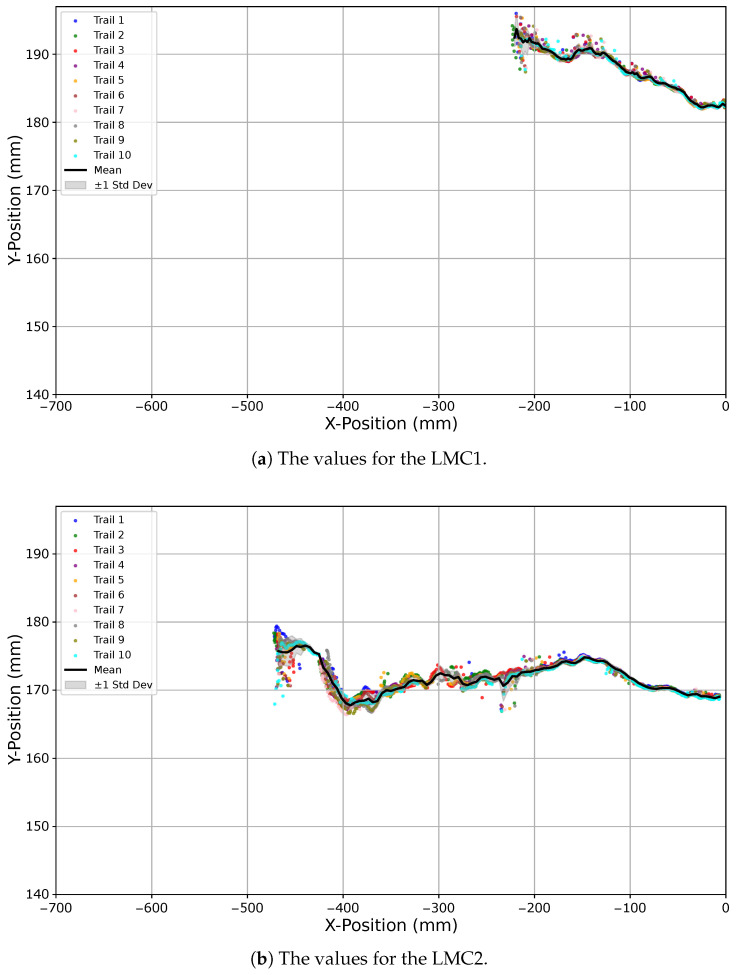
Boundary experiment results for movements into the LMC detection field. Panels show executions with the right hand starting above the controllers and moving outward at 100% robot speed: LMC1 in (**a**) and LMC2 in (**b**). Each dotted line represents one of ten trials, the black line indicates the mean trajectory, and the gray area denotes the standard deviation. Identical axis scaling is used to highlight differences in detection behavior.

The images in Figure 9a,b illustrate the performance of both devices when starting with the hand above the controller and moving outward at 50% robot arm speed. The opposite direction, from outside the field of view towards a position above the respective controller, is shown in Figure 10a for the LMC1 with the left hand and in Figure 10b for the LMC2 with the right hand, both executed at 100% speed. Using the same axis scaling, it becomes evident that the timing and duration of detection strongly depend on the movement direction.

At the edges of the detection domain, both the LMC1 and LMC2 display a noticeable reduction in tracking precision, with deviations in recorded positions becoming more pronounced. This reduction is particularly evident when the hand approaches or exits through oblique or non-perpendicular angles, suggesting that edge tracking is more sensitive to movement direction than tracking within the central detection zone.

Despite these limitations, the devices consistently detect the hand promptly upon entering the detection area, as evidenced by the minimal delay in recognition times. This rapid detection indicates that both systems are well-suited for dynamic applications where quick re-engagement is required after temporary loss of tracking. However, once within the edge region, deviations in positional accuracy and tracking stability become more prominent. This variability underscores the need for careful consideration of edge performance when integrating these devices into systems requiring high precision, such as fine-grained gesture recognition or virtual object manipulation in VR/AR environments.

The extended detection range of the LMC2 up to approximately 0.65 m is particularly relevant for head-mounted configurations, where the sensor is often positioned near the user’s head and hands operate at roughly arm’s length. In such setups, our boundary measurements indicate that LMC2 can maintain continuous tracking over most of the typical reach volume, whereas LMC1 would lose tracking considerably earlier. However, since accuracy still degrades towards the edges of this range, designers of VR/AR interaction techniques should avoid placing high-precision tasks at the limits of the detectable space and instead favor central regions where both accuracy and repeatability are higher.

## 6. Discussion

The results of this study highlight the progress in hand tracking technology represented by the LMC2 compared to its predecessor. Across multiple experiments, the LMC2 consistently demonstrated improved accuracy, robustness, and responsiveness in both static and dynamic tracking scenarios. Notable advancements include the expanded field of view, among others, which enables more reliable tracking within the central detection domain. This can be seen, on the one hand, in the reduction of, e.g., palm-position error from 7.9–9.8 mm (LMC1) to 5.2–5.3 mm (LMC2), and on the other hand in the lower positional variability of 0.44–0.76 mm compared to 1.26–2.18 mm. Furthermore, this is supported by the extended detection range of 666 mm until leaving the detection space and a first-detection range of up to 646 mm when entering the detection range for the LMC2. These improvements can be particularly valuable for VR/AR applications, where fluid and precise hand detection is critical for immersive user experiences, which typically operate at hand-to-sensor distances similar to our measured 40–70 cm range. For many interactive scenarios, such as gaming, training simulations, or everyday HMI tasks, central-region errors in the low millimeter range combined with high repeatability are typically sufficient, as interaction concepts can tolerate small offsets through visual feedback, snapping, or filtering. In contrast, medical scenarios often demand stricter requirements on position accuracy and stability. In these contexts, the observed improvements of LMC2 over LMC1 in the focus region indicate more potential. This should be examined more thoroughly in a domain-specific study, particularly with respect to medical applications and accuracy. Nevertheless, the experiments also revealed challenges that persist, particularly at the edges of the detection area. Both controllers exhibited reduced tracking precision and reliability near their boundaries, with deviations in positional accuracy becoming more pronounced. This limitation is especially relevant in VR/AR environments, where user interactions often extend across the full detection volume, including transitions in and out of the tracking zone. Although the LMC2 demonstrated faster and more consistent re-detection at these boundaries compared to the original LMC1, occasional inaccuracies highlight the need for further refinement to address edge behavior comprehensively. The decrease in spatial accuracy near the edges of the interaction volume observed in our experiments is very similar to what has previously been reported for LMC1. Earlier evaluations likewise described increased positional distortion and reduced stability near the edges of the field of view [1,6,11]. This indicates that the limitations of Leap Motion’s vision setup exist across hardware generations, even though LMC2 reduces absolute error magnitudes and improves repeatability in the central region. Consequently, interaction-design guidelines derived from LMC1 work, such as avoiding precision-critical actions at extreme viewing angles, remain applicable to LMC2. These findings provide a robust benchmark for the applicability evaluation of hand tracking systems for real-world scenarios. The LMC2’s advancements make it particularly well-suited for VR gaming, training simulations, and other interactive applications where responsiveness and ease of use outweigh the need for absolute accuracy and repeatability. However, for applications that rely on very high accuracy, including surgical assistance, medical imaging, or precise industrial tasks, further technological improvements may be necessary. The analysis also emphasizes the broader implications for the development of touchless HMI. As VR/AR technologies continue to evolve, the demand for seamless and intuitive interaction mechanisms will only grow. Devices like the LMC2 represent a significant step toward meeting these expectations, offering improved usability and versatility in applications ranging from entertainment to professional training.

Taken together, the similarity of the spatial error structure between LMC1 and LMC2, combined with the reduced central errors and extended detection range of LMC2, suggests that qualitative conclusions drawn from earlier LMC1-based studies can remain valid, while our results provide the quantitative scaling factors needed to reinterpret those findings for the newer hardware generation.

Future studies should focus on addressing the remaining challenges identified in this work, particularly edge-case performance and the tracking of high-speed or complex movements. Furthermore, the usage of 3D-printed hands cannot represent different anatomy types, pose variability, skin reflectance and occlusions. Therefore, experiments with human participants are a logical next step to examine these limitations in real-world conditions. Additionally, integrating complementary sensors or leveraging machine learning to predict and correct tracking inaccuracies may further enhance the capabilities of hand tracking systems like the LMC2. It still remains to be tested how the comparison looks under different ambient lighting conditions and with more noise. A comparison with integrated HMD trackers is also still missing, as well as a closer investigation of dual-LMC2 setups to understand their effects on the edge regions. By addressing these areas, hand tracking technology can continue to close the gap between current capabilities and the demands of next-generation VR/AR applications.

## Figures and Tables

**Table 1:** Overview of the average accuracy and repeatability of the positions approached in the field of view experiment (all 75 points) measured with either left or right hand.

	LMC1 (Left)	LMC2 (Left)	LMC1 (Right)	LMC2 (Right)
	Accuracy (mm)			
Thumb	19.51	19.14	23.32	15.21
Index	17.16	17.97	17.71	18.09
Middle	21.93	20.21	14.52	22.01
Ring	21.92	18.00	16.84	19.64
Pinky	23.46	18.09	14.92	19.65
Palm	17.07	11.70	13.40	10.96
	**Repeatability (mm)**			
Thumb	3.85	1.76	4.06	1.42
Index	3.16	1.54	3.32	1.64
Middle	3.23	1.92	2.82	1.44
Ring	3.30	1.57	2.84	1.17
Pinky	3.21	1.62	2.31	1.30
Palm	2.82	1.22	1.60	0.96

**Table 2:** Overview of the average accuracy and repeatability of the positions approached in the field of view experiment for a reduced cubic grid of 27 points measured with either left or right hand.

	LMC1 (Left)	LMC2 (Left)	LMC1 (Right)	LMC2 (Right)
	Accuracy (mm)			
Thumb	10.59	9.93	12.02	4.93
Index	7.52	8.16	9.59	7.01
Middle	13.34	7.34	9.68	8.92
Ring	14.18	5.87	8.68	7.59
Pinky	14.48	7.24	9.41	8.40
Palm	9.78	5.30	7.90	5.24
	**Repeatability (mm)**			
Thumb	2.85	1.34	1.96	0.32
Index	2.38	1.11	1.65	0.43
Middle	2.58	1.00	2.28	0.41
Ring	2.55	1.05	1.35	0.49
Pinky	2.40	1.03	1.38	0.43
Palm	2.18	0.76	1.26	0.44

**Table 3:** Number of failed hand detections (out of 750 measurements) and corresponding success rates, given as decimal fractions (e.g., 0.95 corresponds to 95%), for both controllers (LMC1 and LMC2), evaluated separately for the left and right hand as well as for two regions of interest: the full field of view (75 points) and the central focus area above the sensor (27 points).

		# Failures (Out of 750)		Success Rate	
		75 Points	27 Points	75 Points	27 Points
LMC1	Left	80	0	0.893	1.000
	Right	111	0	0.852	1.000
LMC2	Left	44	0	0.941	1.000
	Right	24	0	0.968	1.000

**Table 4:** Boundary experiments (Figure 3) moving to the outside for both hands with 50% and 100% robot arm speed. The values represent the longest detection range for x-axis movement out of ten repetitions, as well as the mean value.

			Left Hand		Right Hand	
		Speed	50%	100%	50%	100%
	Metric					
LMC1	Maximum range (mm)		249.18	275.37	258.76	266.21
	Mean maximum range (mm)		242.32	251.27	256.66	261.86
LMC2	Maximum range (mm)		436.12	555.10	645.29	666.28
	Mean maximum range (mm)		394.70	456.56	594.26	634.46

**Table 5:** Boundary experiments (Figure 3) moving above the LMCs for both hands with 50% and 100% robot arm speed. The values represent the first detection entering the field of view for x-axis movement and the mean value, based on ten repetitions.

			Left Hand		Right Hand	
		Speed	50%	100%	50%	100%
	Metric					
LMC1	Maximum range (mm)		230.17	223.17	176.91	176.22
	Mean maximum range (mm)		224.20	215.96	161.17	156.03
LMC2	Maximum range (mm)		269.02	268.52	646.58	472.33
	Mean maximum range (mm)		263.76	263.95	504.31	469.38

## Data Availability

The data presented in this study are available on request from the corresponding author.

## Abbreviations

The following abbreviations are used in this manuscript:

VR	Virtual reality
AR	augmented reality
HMI	human–machine interaction
LMC	Leap Motion Controller
LMC1	Leap Motion Controller 1
LMC2	Leap Motion Controller 2
SVD	Singular value decomposition
CCD	Charge-Coupled Device
